# Microwave-Driven Exfoliation of Bulk 2H-MoS_2_ after Acetonitrile Prewetting Produces Large-Area Ultrathin Flakes with Exceptionally High Yield

**DOI:** 10.1021/acsnano.3c00280

**Published:** 2023-03-14

**Authors:** Ramiro Quirós-Ovies, María Laborda, Natalia Martín Sabanés, Lucía Martín-Pérez, Sara Moreno-Da Silva, Enrique Burzurí, Víctor Sebastian, Emilio M. Pérez, Jesús Santamaría

**Affiliations:** † Instituto de Nanociencia y Materiales de Aragón (INMA), CSIC-Universidad de Zaragoza, Zaragoza 50009, Spain; ‡ IMDEA Nanociencia C/Faraday 9 Ciudad Universitaria de Cantoblanco, 28049 Madrid, Spain; § Departamento de Física de la Materia Condensada and Condensed Matter Physics Center (IFIMAC), Universidad Autónoma de Madrid, 28049 Madrid, Spain; ∥ Department of Chemical and Environmental Engineering Universidad de Zaragoza Campus Rio Ebro, 50018 Zaragoza, Spain; ⊥ Networking Research Center on Bioengineering, Biomaterials and Nanomedicine (CIBER-BBN), 28029 Madrid, Spain; # Laboratorio de Microscopías Avanzadas, Universidad de Zaragoza, 50018 Zaragoza, Spain; ○ Instituto Universitario de Ciencia de Materiales “Nicolás Cabrera” (INC), Universidad Autónoma de Madrid, E-28049 Madrid, Spain

**Keywords:** molybdenum disulfide, transition-metal dichalcogenides (TMDCs), microwave-driven exfoliation, large-area ultrathin flakes, high lateral size, high yield process

## Abstract

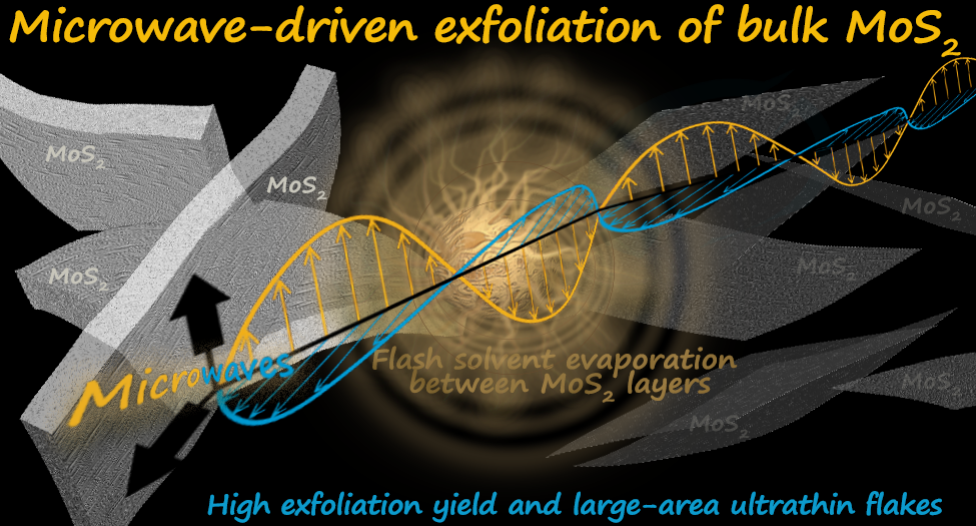

2D materials display exciting properties in numerous fields, but the development of applications is hindered by the low yields, high processing times, and impaired quality of current exfoliation methods. In this work we have used the excellent MW absorption properties of MoS_2_ to induce a fast heating that produces the near-instantaneous evaporation of an adsorbed, low boiling point solvent. The sudden evaporation creates an internal pressure that separates the MoS_2_ layers with high efficiency, and these are kept separated by the action of the dispersion solvent. Our fast method (90 s) gives high yields (47% at 0.2 mg/mL, 35% at 1 mg/mL) of highly exfoliated material (90% under 4 layers), large area (up to several μm^2^), and excellent quality (no significant MoO_3_ detected).

Transition metal dichalcogenides (TMDCs) are an emerging class of layered materials with highly interesting electronic and photonic properties.^1−4^ They are based on a MX_2_ structure where M is a transition metal (Ti, Nb, Ta, Mo, W, etc.) and X is a chalcogen (S, Se, Te), forming three-atom layers that are linked together through weak van der Waals forces. Among TMDCs, the thermodynamically favored polytype of MoS_2_, 2H-MoS_2_, is attracting the most attention, due to its appealing electronic properties, which depend crucially on the number of stacked layers.^5−8^ Bulk 2H-MoS_2_ presents an indirect bandgap of 1.23 eV, while monolayer MoS_2_ exhibits a direct bandgap of 1.90 eV.^7,9,10^ Thin MoS_2_ flakes can be obtained by exfoliation from the bulk or by synthetic methods. Among exfoliation methods, mechanical exfoliation produces large area MoS_2_ flakes of very high quality, but it is inherently very low yielding.^11−14^ In contrast, liquid phase exfoliation (LPE) methods typically involve ion intercalation,^15−18^ hydrothermal intercalation,^19^ and/or long treatments with ultrasound,^20−26^ followed by centrifugation at >10000 rpm to isolate the deeply exfoliated fractions present in the supernatant. Irrespective of the method, liquid phase exfoliation procedures in general give very low yields of monolayer and few-layer MoS_2_ (see Table 1), and this only after long and often complex processing procedures. Sometimes, suspensions containing large amounts of MoS_2_ flakes can be obtained, but with broad thickness distributions.^21,23−28^ Iterative purification steps based on staged centrifugations can yield suspensions enriched in monolayer and few-layered, 2H-MoS_2_, but this leads to low yields and, since they are based on sedimentation rates, typically small area flakes.^21,23,28^

**Table 1:** Brief Summary of Previous Attempts to Exfoliate 2H-MoS_2_ Polytype

Exfoliation method	Time	Scalability	Yield	Lateral size	Number of layers	Comments	ref
Sonication	>3 h	Possible	1–10%	>200 nm	2–20	Heterogeneous heights, small flakes and, low yield	(20−25)
Chemical (ion intercalation)	>1 day	Possible	80%	<1 μm	1–5	1T-MoS_2_ is obtained. Difficulties washing the intercalation agents, e.g., *n*-BuLi.	(15−18)
Mechanical (Scotch tape)	-	Not possible	1–10%	Up to mm	1–10	Extremely slow, impractical	(11−14)
Shearing (blender/homogeneizer)	2 h	Possible	<10%	40–220 nm	2–12	Damaged flakes, long process, heterogeneous distribution of flake size	(26)
Ball-milling	>10 h	Only batch processing	10–90%	50–150 nm	2–4	Time-consuming, lower yields at higher loads, small lateral sizes	(29)
Hydrothermal	1–2 h	Possible	10%	200 nm – 1 μm	4–10	Time consuming	(19)
Electrochemical	2 h	Crystalline MoS_2_ is required	Not mentioned	1–50 μm	1–2	Time consuming, conductive crystal required, low yields	(30)
Fluid dynamics	1 h	Possible	80%	1–2 μm	1–2	Mixtures of 1T-MoS_2_ and 2H-MoS_2_ are obtained.	(32)
Laser-assisted	10 min	Possible but complex	Not mentioned	50–150 nm	20–40	Obtention of 1T-MoS_2_, thick and small flakes	(33)
Compressed flow exfoliation	2 min	Achieved	10%	200–300 nm	3–4	Extremely fast but low lateral size-thickness ratio	(37)
Wet grinding plus MW- exfoliation	1 h	Grinding stage should be made continuous	25%	1–2 μm	3–4	High yields given correspond to thicker material. Not particularly fast.	(41, 42)
This method	90 s	Possible	47%	1–4 μm	1–4	Fastest method to date, scalable, high yield, large but thin flakes	

From the above, it can be concluded that a significant proportion of ultrathin 2H-MoS_2_ flakes by LPE can only be obtained at the expense of very low yields. Also, the long sonication processes that are the norm not only represent a significant processing cost but also often result in a lower quality of the exfoliated material in terms of chemical (e.g., oxidation state) and morphological (lateral size and edge roughness) features. In an attempt to improve the yield of highly exfoliated materials, other techniques have been tested. Thus, ball milling in the presence of sodium cholate gave good results in terms of yield, but required extremely long times (up to 20 h), and the severity of the mechanical treatment reduced the lateral size of the flakes to values under 200 nm.^29^ Electrochemical exfoliation is another example of a system with intrinsic scalability problems, requiring MoS_2_ to be present as the anode of the cell.^30^ It gave a similar material to chemically exfoliated MoS_2_, with a lateral size between 1 and 50 μm.^31^ Other methods as fluid dynamics exfoliation^32^ achieve extremely thin and large lateral-sized flakes, along with high yields. However, as in the case of laser-assisted^33^ and chemical exfoliation, the polytype obtained by these processes is the metallic 1T-MoS_2_, and its properties and applications^34^ are utterly different from the ones of semiconducting 2H-MoS_2_.^8,35,36^ Extremely fast and high-yielding methods have already been successfully studied. As an exceptional example, compressed flow exfoliation gives 2H-MoS2 in only 2 s with yields up to 10%.^37^ The lateral size of these flakes comprises 200–300 nm, comparable to the material obtained by standard sonication. Microwaves have also been used as an exfoliation tool for MoS_2_, as well as for other 2D materials, mainly graphite.^38−40^ In general, microwave-aided processes make use of the fast and volumetric heating associated with microwaves and have demonstrated significant improvements regarding the yield of exfoliated material, and also some progress regarding the maintenance of desirable properties, such as lateral size, that is strongly reduced under prolonged sonication treatments. However, so far there is not a clear recipe for microwave-aided exfoliation of MoS_2_, and some of the methods reported present surprising requirements. Thus, for instance, Liu and co-workers^41^ used microwave heating to exfoliate several 2D materials (MoS_2_ among them) and showed examples of well exfoliated material, down to few-layer nanosheets, although yield values were not quoted. They attributed their success to the bursting pressure caused by the evaporation of the solvent that was previously intercalated into the interlayer space of the material. However, the authors claimed that for the success of the treatment it was necessary that the bulk materials to be exfoliated were previously bonded to a SiO_2_/Si surface by means of adhesive tape. Otherwise, they contended, the random motion of the bulk powder would strongly reduce the efficiency of the exfoliation. Wu et al.,^42^ on the other hand, again used a microwave-aided exfoliation in a two-step procedure but needed to employ a previous stage consisting of wet grinding for a minimum of 10 min (otherwise the yield dropped dramatically), followed by a microwave-aided exfoliation for 30–60 min. Interestingly, the authors tested different solvents (organic solvents and ionic liquids), that had to meet two essential requisites: disperse well the materials and efficiently absorb microwave energy. They obtained high yields (up to 30% for MoS_2_) and good lateral sizes (0.5–3 μm for MoS_2_), although again they faced stringent limitations. Not only was a first wet grinding step needed, but there were also important limitations regarding the concentration of the solutions that could be processed (under 3.5 mg/mL), which in turn limited the amount that could be obtained and the scalability of the process.

Here, we present a very fast (90 s exfoliation time) and high-yielding (>45% at 0.2 mg/mL) microwave-assisted methodology for the LPE of MoS_2_ that produces flakes of large area (up to several μm^2^), with a high proportion of exfoliated material (90% of flakes thickness <5 nm) and a quality (no significant MoO_3_ detected) comparable to that obtained by mechanical exfoliation. The obtention of high aspect ratio flakes has attracted notable attention as it has proved to be extremely difficult to achieve a material that exhibits large lateral dimension while being an ultrathin material.^43^ In addition, we remove many of the requirements stated in previous work: there is no need to immobilize the bulk material, of previous wet grinding or of using solvents able to absorb microwaves themselves. The method developed in this work takes advantage of the high MW absorption capabilities of MoS_2_ to achieve ultrafast heating of the bulk material. This rapid temperature rise produces a sudden vaporization of any solvent that had been previously introduced between MoS_2_ layers.

## Results and Discussion

**Scheme 1:**
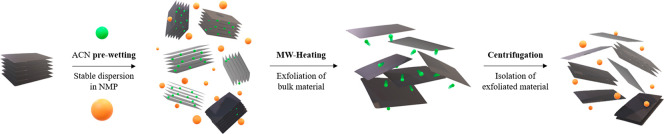
Scheme of the Experimental Procedure for the MW-Assisted Exfoliation Using the Combination of Two Solvents

As mentioned above, the prewetting stage before microwave irradiation to wet a low boiling point solvent is key to the success of the exfoliation. The solvent (prewetting solvent) used should be able to wet the material but (unlike the requirements of previous works) does not need to absorb microwaves itself, since we rely on the MoS_2_ material for efficient MW heating. If the intercalated solvent does not perform well at stabilizing exfoliated MoS_2_, a second solvent able to disperse exfoliated MoS_2_ must added to the system. Again, this solvent does not need to absorb MW. For the prewetting stage different solvents were tested (see complete list in Table SI 1, Supporting Information). Acetone, THF, and acetonitrile (ACN) provided an excellent exfoliation yield (Table SI 1). Finally, we have chosen acetonitrile, a solvent that is known to wet MoS_2_ very well, with contact angles <20°,^44^ and is sufficiently small to intercalate between the bulk MoS_2_ layers during prewetting.^45^ It is not a strong MW absorber (its dielectric loss is about 4 times smaller than that of water), but as already stated, this does not matter because MoS_2_ itself will be heated preferentially. More importantly, acetonitrile has a low boiling point (82 °C), and its vapor pressure curve is steep, from 89.4 Torr at 25 °C to 368 at 60 °C. Since ACN performs poorly at stabilizing MoS_2_ suspensions, after prewetting a larger quantity of a second suitable solvent was added, and bulk MoS_2_ was dispersed into it. Several dispersion solvents were tested (Table SI 2, Supporting Information), and the best results were obtained with *N*-methylpyrrolidone (NMP) and acetone. NMP is optimal for the stabilization of MoS_2_ colloidal suspensions and has a high boiling point (202 °C), so it will remain liquid, while MW-driven exfoliation takes place with the sudden evaporation of ACN trapped between MoS_2_ layers. As soon as the MoS_2_ layers are exfoliated, the colloidal suspension is stabilized by NMP, preventing reaggregation (Scheme 1).^46^

It is essential that MW heating of MoS_2_ takes place efficiently and rapidly, so that the whole process (pressure buildup between MoS_2_ layers followed by ACN evaporation and cavitation) that causes the efficient separation of the layers takes place before ACN has been able to diffuse out of the bulk material.

**Figure 1:**
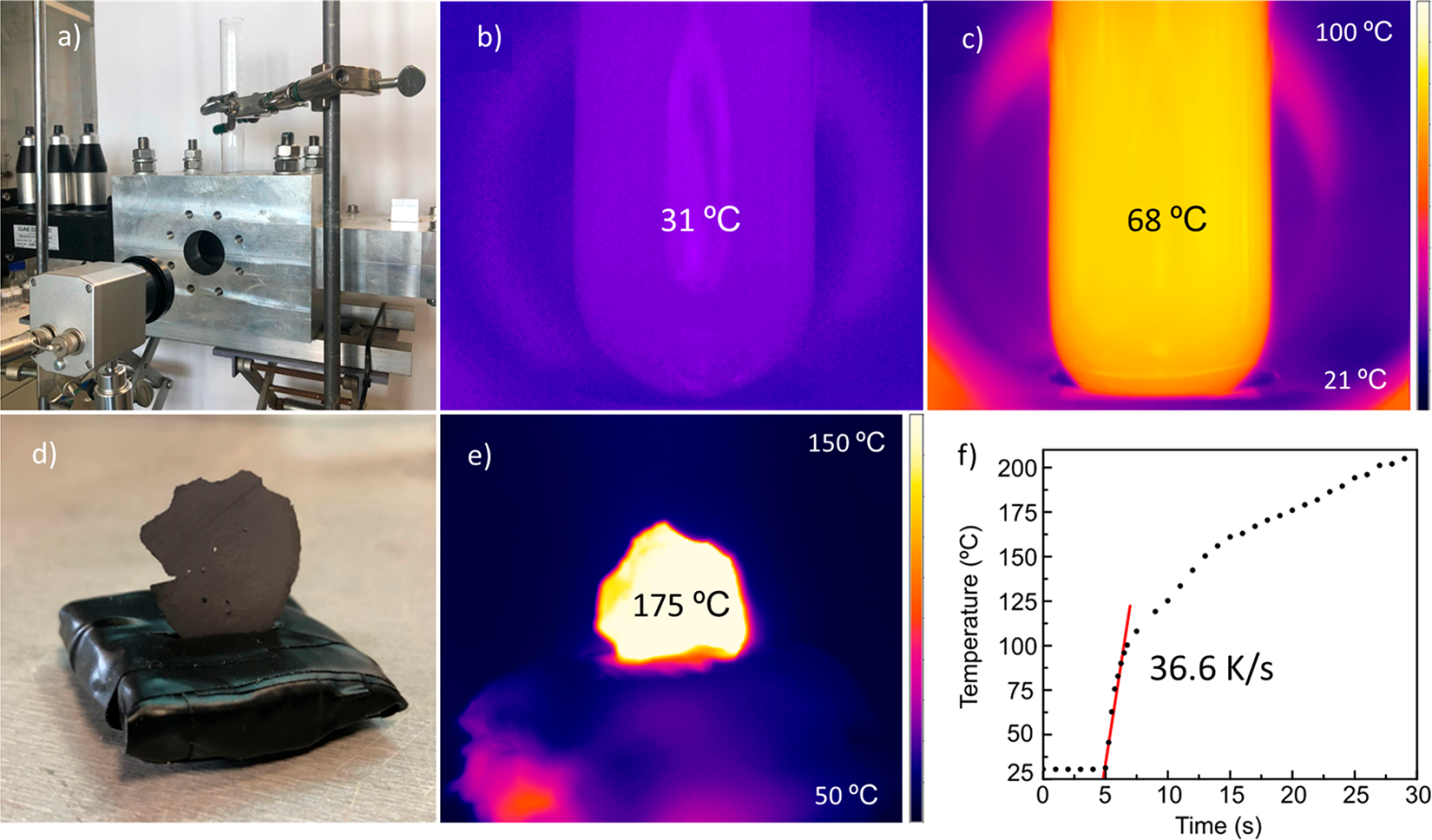
(a) Experimental setup for measuring the temperature evolution of MoS_2_ under microwave heating, with a thermographic camera facing a window in the MW waveguide. (b,c) Thermographic images after 25 s of MW heating at 30 W of (b) test tube containing 30 mL of NMP and 0.5 mL of ACN (corresponding to the volume used for prewetting) and (c) the same volume of a 1 mg/mL dispersion of bulk MoS_2_ in NMP. (d) Pressed bulk MoS_2_ slab supported onto a polyurethane base, before MW heating. (e) Thermographic image of a dry MoS_2_ slab after 25 s of MW heating. (f) Temperature evolution of the dry slab under MW heating.

To demonstrate the fast intrinsic heating of bulk MoS_2_ under a MW field, we carried out experiments in which the temperature evolution of dry MoS_2_ slabs and of suspensions of the solvents used with and without MoS_2_ was followed in real time. Figure 1b and c compare the heating of the solvents used without and with the presence of bulk MoS_2_ at 1 mg/mL concentration. It can be seen that without MoS_2_ in the solution, the absorption of energy is strongly diminished. Then, a dry pressed MoS_2_ slab was subjected to MW heating. It can be seen that a very fast heating rate is obtained, with temperature increase rates around 37 K/s. These results show that MoS_2_ is an excellent MW susceptor, able to capture MW energy efficiently. The fast temperature increase of bulk MoS_2_ would then be transferred to the ACN intercalated between the MoS_2_ layers (Figure 1d–f), producing its sudden evaporation and the exfoliation of the material as a consequence of the resulting internal pressure.

**Figure 2:**
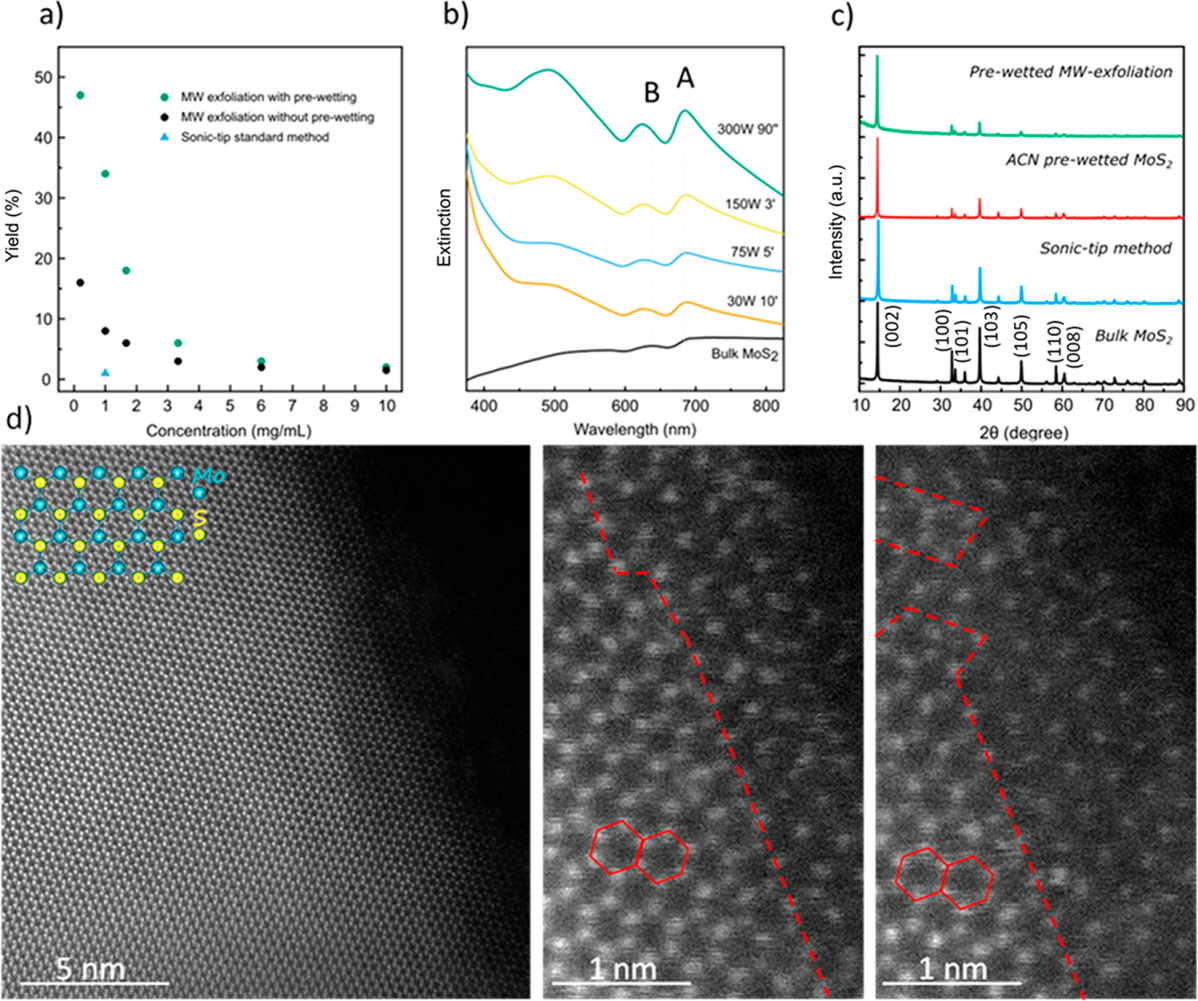
(a) Yield of the NMP-ACN exfoliated samples related to the initial concentration of bulk MoS_2_. (b) UV–vis spectra of various MoS_2_ samples, exfoliated with different power-time conditions, compared to bulk MoS_2_ (black). (c) Comparison of the XRD structure peaks disappearance of bulk MoS_2_ (black), sonic-tip exfoliated MoS_2_ (blue), ACN prewetted MoS_2_ (red), and prewetted MW-exfoliated MoS_2_ (green). (d) HAADF-STEM images with atomic resolution of MW exfoliated MoS_2_. Inset, schematic illustration of the structure of MoS_2_ (2H phase). Red dashed line delimits the area where vacancy defects are located.

As shown in Tables SI 1, SI 2 and Figures SI 3–4 in the Supporting Information, few-layered MoS_2_ could be prepared by microwave-assisted heating of bulk MoS_2_ after prewetting with different solvents followed by dispersion in the same or a different solvent, although the best results were obtained with ACN as the prewetting agent and NMP as the dispersion solvent. Specifically, 1 mL of ACN was allowed to wet the layers of the bulk material for 5 to 10 min. Subsequently, 30 mL of NMP were added, and the sample was subjected to microwave irradiation. Interestingly, MW-driven exfoliation without prewetting could still take place, but the improvement obtained by prewetting with a volatile solvent before irradiation is obviously remarkable from the quantitative evaluation of the yield of the exfoliation process, roughly tripling the yields obtained without prewetting and being some 50 times higher than the results of ultrasound-based exfoliation (Figure 2a and Figure SI 1). It must be noted, however, that comparing the yields obtained in different works is sometimes difficult due to the lack of a clear definition of the conditions used. In this work we calculated yields by filtering the supernatant after centrifugation at 448 g for 20 min, using a 0.2 μm filter, and expressed the yield as a percentage ratio of the amount of material retained in the filter to the starting mass of MoS_2_. Figure 2a also shows that the yield decreases as MoS_2_ concentration increases. This is due to the high MW absorption capacity of MoS_2_, that creates a shielding effect that reduces the MW intensity reaching inner fluid volume elements. Considering these data, we selected 1 mg/mL as a reference concentration for subsequent experiments (yield = 34%). Although even better yields could be obtained at 0.2 mg/mL (yield = 47%), such low concentrations would be impractical for larger scale exfoliations.

NMP dispersions of ACN-wetted MoS_2_ samples were subjected to different combinations of irradiation power and time (see Table SI 1) using the extinction spectra of the suspensions as obtained after 1 centrifugation cycle (448 g, 20 min), as first indication of exfoliation (Figure 2b). A clear increase of the total absorbance and of the relative absorbance of the excitonic transitions with increasing irradiation power can be observed (Figure 2b). This improvement observed with high MW field intensity cannot be balanced by an increase in irradiation time at lower power. For example, irradiating at 300 W for just 90 s results in very well-defined and intense excitonic bands at 684 and 624 nm for excitons A and B, respectively (Figure 2b). In comparison, the suspension irradiated at 30 W for 10 min affords much lower total absorbance and a clearly dispersion-dominated extinction spectra, with the excitonic peaks significantly red-shifted to 691 and 631 nm (Figure 2b). These results are consistent with our hypothesis: the exfoliation process is favored by a more intense irradiation, since this induces a fast local temperature increase of the MoS_2_ nanosheets and a rapid heat transfer to the liquid confined among the layers, whose pressure increases leading to exfoliation. From the data summarized in Tables SI 1, SI 2 and Figure SI 2, we settled on 300 W and 90 s as optimized irradiation power and time.

The characterization of the MoS_2_ samples exfoliated using these optimized conditions allowed us to demonstrate the high efficiency of the exfoliation process. According to the powder X-ray diffraction data (XRD), a progressive disappearance of MoS_2_ crystalline signals is observed in the series bulk/prewetted/MW-exfoliated (Figure 2c). This may be attributed to the initial separation of the layers due to acetonitrile intercalation and then to the complete disconnection of the layers as MW-induced evaporation of the solvent exfoliates the sheets. The XRD data of our exfoliated sample (Figure 2c) is dominated by the in-plane diffraction at 2θ = 15° and shows complete depletion of all out-of-plane diffractions.^31^

Atomic-resolution HAADF-STEM imaging was performed on the MW exfoliated MoS_2_ to evaluate the introduction of sulfur vacancies during the exfoliation procedure.^47^Figure 2d depicts the atomic structure of the MW exfoliated MoS_2_, where the slab of hexagonal Mo lattice is sandwiched between two layers of hexagonally packed S. Due to the HAADF detector, Mo atoms show brighter contrast than S atoms because of the atomic number difference. However, Figure 2d shows that the contrast is almost comparable between Mo and S sites, despite the lower atomic number of S. This phenomenon can be rationalized because the signal from the S sites is enhanced due to overlap of two sulfur atoms along the electron beam direction, which is typical from 2H-MoS_2_ atomic arrangement.^48−50^ The presence of few vacancy defects (most likely S vacancies) was also observed at the edge region (Figure 2d). This is a consequence of the mechanical cleavage produced by the sudden vaporization of the solvent (acetonitrile) boosted by the microwaves and that was previously introduced between MoS_2_ layers.^47^

**Figure 3:**
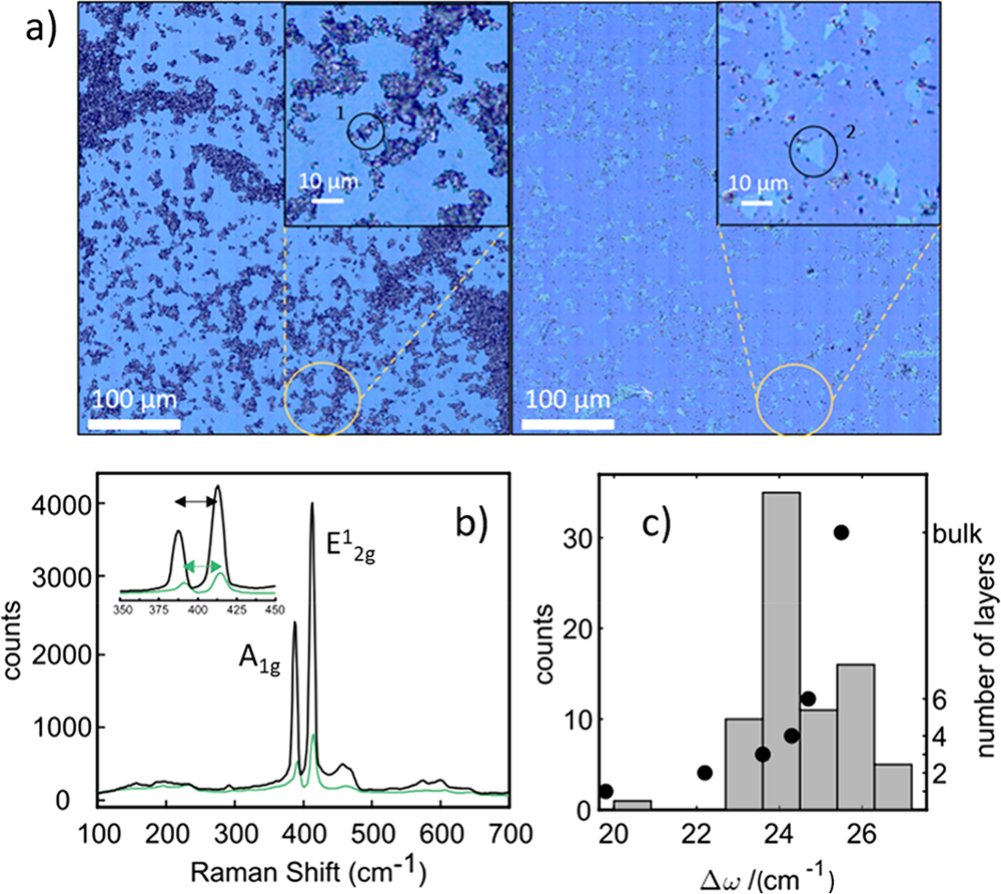
(a) Optical image of a representative area of the sample, showing MoS_2_ flakes of different sizes and thicknesses together with bulk MoS_2_ powder (gray). (b) Raman spectra of bulk (black) and a thin flake taken at locations marked in a). (c) Bar histogram of Δω = ω(A_1g_) – ω(E_2g_^1^) in the Raman spectra measured over several different flakes in the sample (left *y*-axis). The relation between number of MoS_2_ layers and Δω according to refs (56 and 58) is included in black circles (right *y*-axis).

The exfoliated flakes were observed under an optical microscope and Raman spectroscopy (λ_exc_ = 633 nm) recorded and compared to the bulk material. Samples were deposited on SiO_2_ by drop-casting a single drop of the exfoliated MoS_2_ dispersion in ACN. This dispersion was prepared by ultracentrifuging the samples in NMP, discarding the supernatant and redispersing in ACN (the process was repeated three times, in order to remove the majority of the NMP). We note that the flakes obtained are apparently very uniform in height, as reflected in their uniform contrast (Figure 3a).^32^ The flakes are also remarkably large in lateral size, with most flakes showing lateral sizes in the μm scale (1.2 μm ± 0.7 μm) (Figure SI 5). This is directly comparable to the lateral size of the bulk crystals (Figure SI 5), which implies that there is no (or very little) lateral size reduction during the exfoliation process. It is important to note here that the typical lateral size of flakes obtained from other LPE methods is in the tens or few hundreds of nm range.^22,25,51−55^ The first conclusion from the Raman data is that there is no damage to the MoS_2_ structure during the exfoliation process. The difference in Raman shift of the in-plane E_2g_ (ca. 383 cm^–1^ in the bulk) and the out-of-plane A_1g_ (ca. 410 nm in the bulk) modes afforded direct information on the thickness of the flakes.^56−58^ Raman spectra were recorded in several different flakes (several spectra per flake taken depending on size). The optical images in Figure 3a and Figures SI 8–9 reveal thin flakes of different sizes and thicknesses over the whole sample, together with bulk powder (Figures SI 6–7). The difference between the A_1g_ and E^1^_2g_ modes (Figure 3b) can be used to resolve the number of layers of the exfoliated material, where bulk material presents Δω of 26 cm^–1^, while thin flakes present smaller values of Δω, as plotted in Figure 3c (black circles).^59,60^ The distribution of Δω plotted in Figure 3c would therefore indicate that a majority of flakes in our sample belong to the 3 to 4 layers range.

**Figure 4:**
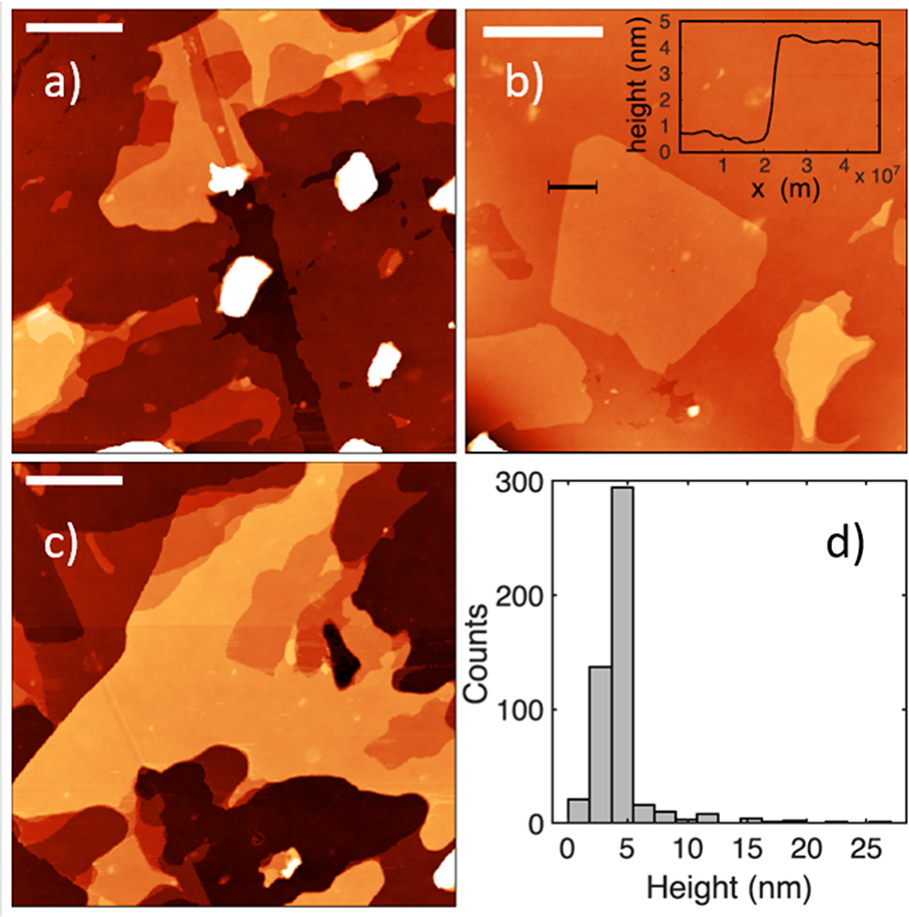
(a–c) Representative AFM images from different areas of the sample (scale bar 1 μm). Inset in (b) shows an example of a height profile. (d) Histogram of recovered heights of more than 30 flakes analyzed from different images.

We also used high-resolution electron microscopy (EM) analysis and atomic force microscopy (AFM) to directly measure the thickness of the exfoliated flakes on the same samples (Figure 4 and Figure SI 10–12). EM images clearly depict the differences between bulk (Figure SI 10) and exfoliated flakes (Figure SI 11), observing exfoliated flakes with less than 5 layers, in agreement with Raman analysis. AFM imaging was performed on different areas of the sample and representative images are included in Figure 4a–c. Flat MoS_2_ flakes (flatness is evident in the example height profile included in inset of Figure 4b) are present all over the sample together with bulk MoS_2_ specimens of heights in the μm range (Figure 4a, white spots). Flakes range from hundreds of nm to several μm in lateral size (in agreement with SEM measurements, Figure SI 5) and often present several heights (different number of MoS_2_ layers within a flake). The results presented in Figure 4d correspond to the height analysis of over 450 flakes found in 12 different sample areas. The distribution of heights shows that the vast majority of flakes are on the 3–5 nm height range (Figure 4 and Table SI 3).

We obtain a very narrow distribution of thicknesses, centered at 3.9 nm and with >90% of the flakes with thicknesses under 6 nm. Although directly correlating thickness as measured in AFM with the number of layers of each flake can be complicated by residual solvent layers, Backes et al. have described an apparent thickness of 1.9 nm per MoS_2_ layer.^40^ Using this value, our AFM data show that our exfoliated flakes are predominantly 1, 2, 3, and 4 layers of MoS_2_, with hardly any flakes of thickness >5 layers (Figure 4d and Table SI 3). In other reports^53^ 1.11 nm is quoted as the apparent height per layer in AFM imaging. Even with this lower value, the average thickness of exfoliated material would be under 4 layers, which is perfectly consistent with the Raman and EM data described above.

Remarkably, we observe little reaggregation following MW-driven exfoliation in any of the characterization methods, highlighting the role of residual NMP as a coordinating solvent (Figure SI 13). This observation holds true for all the surface deposition techniques we have tried (drop-casting in NMP and in ACN, with and without filtering the samples; Langmuir–Blodgett films preparation) but is unexpectedly conserved even for dry powder. Finally, XPS confirms the existence of only one polytype of the exfoliated material (semiconductive 2H-MoS_2_, with no trace of the metallic 1T phase), in agreement with electron microscopy and XRD analyses (Figure SI 14). Also, Mo (VI) was not observed after MW-assisted exfoliation, implying that the exfoliated samples were not oxidized. Mo/S stoichiometry was also examined within XPS data, obtaining a ratio S–Mo of 2.09 by correlating both areas. Hereby it is confirmed that the exfoliated material exhibits a MoS_2_ composition.

As a proof-of-concept evaluation for a final-end use of the MW-exfoliated material the electrical properties of exfoliated MoS_2_ flakes are explored in solid-state electronic devices. MoS_2_ flakes are placed into metallic electrodes directly from NMP solution by dielectrophoresis (Figure SI 15).^61^ The current–voltage characteristics show that MoS_2_ retains the semiconducting-like behavior, with resistance values in agreement with those observed in similar devices made of thin MoS_2_ flakes (Figure SI 15a,b).^62,63^ See Supporting Information for full details of device preparation and electron transport measurements.

## Conclusions

In conclusion, a truly few-layered material that exhibits micron-order lateral size of the flakes can be obtained with high yield using a fast two-solvent microwave-assisted exfoliation. The excellent MW absorbing capabilities of MoS_2_ and the symbiotic behavior of acetonitrile (wetting, high volatility solvent) and *N*-methyl pyrrolidone (high ability to disperse exfoliated material) produce a highly efficient separation of the layers with minimal damage to the nanosheets and keeping largely intact their pristine lateral size. The exfoliated material comprises mainly sheets from 3 to 5 layers, with an average thickness of 3.9 nm. The yield of the process is roughly 50 times greater than ultrasonication exfoliation methods and gives a material quality comparable to mechanical exfoliation, whose flakes are similar to those obtained by this method but with an incomparably higher yield. In summary, this method takes the best of both worlds (mechanical and liquid phase exfoliation), improving the results of each case in terms of thickness, lateral size, yield, and processing time. It has been applied to MoS_2_, but it could be used to exfoliate any material with high MW absorption capabilities, being a versatile approach in the area of emerging 2D materials.

## Methods

### Materials

MoS_2_ powder (99%), acetonitrile, isopropyl alcohol, tetrahydrofuran, dichloromethane, and chloroform were purchased from Merck; *N*-Methylmaleimide (>98%) was obtained from TCI Europe; water was obtained from a Milli-Q filtration station (“Type 1” ultrapure water; resistivity: 18.2 MΩ·cm at 25 °C).

### Exfoliation Method

Initially, 30 mg of bulk MoS_2_ were prewetted with 0.5 mL of ACN for 5 min, until the solvent is almost evaporated. Once the ACN has wetted the MoS_2_ layers, 30 mL of *N*-methyl-2-pyrrolidone (NMP) were added. After sonicating the sample 1 min to disperse the material, the dispersion was transferred to a glass reactor and introduced into the MW cavity (CEM Discovery monomodal MW unit). The material was heated under the desired power for 90 min under vigorous stirring, then allowed to cool down and transferred to a centrifuge tube. The mixture was centrifuged at 2000 rpm for 20 min (AllegraX-15R Beck-man Coulter centrifuge, FX6100 rotor, 208 C). Then, the supernatant (olive-color) was separated from the nonexfoliated MoS_2_ by decanting and filtered through a 0.2 μm membrane (Omnipore PTFE membrane filter). The membrane with the retained exfoliated MoS_2_ was dispersed in acetonitrile and filtered again. This redispersion process was repeated three times to remove all the NMP. Finally, the powder was weighed and characterized by a battery of techniques.

We only considered the mass obtained after centrifuging and filtering to calculate the yield of the process.

### Solid-State Device Fabrication

The source–drain electrodes are fabricated via laser maskless optical lithography and thermal evaporation of Cr/Au (5/80 nm) on a highly doped silicon substrate capped with a 300-nm-thick insulating SiO_2_ layer, used as a common back-gate electrode. Initially, Si/SiO_2_ wafers are cleaned using *i-*PrOH and acetone to remove any traces of organic, ionic, and metallic impurities. Then, AZ1505 positive photoresist is spin coated at 5000 rpm for 1 min onto the surface followed by baking at 90 °C for 1 min to form a 450 nm resist layer. The electrodes and contact pads are defined by exposing the surface to UV light using a Heidelberg Instruments DWL66 fs laser writer of 405 nm (h-line) with 300 mJ/cm^2^ dose. The pattern is subsequently developed with AZ-351B. Thereafter, 5 nm Cr and 80 nm Au layers are deposited using Ecovac e-beam evaporation by Angstrom Engineering. A lift-off process in acetone/*i-*PrOH/deionized water removes the excess metallic material. The individual finger-shaped electrodes are connected to Au pads that allow electrical contact in an electrical probe station. The size of the gap created between a pair of electrodes is 1 μm with minor variations from device to device. The devices are annealed at 300 °C for 8 h after the fabrication.

### UV–vis Spectroscopy

UV–vis spectroscopy was performed using a UV-670 UV–vis spectrophotometer from JASCO and 1 cm light-path quartz cells. All measures were done from 850 to 350 nm with a halogen lamp and a 1 nm/s scan speed.

### Raman Spectroscopy

Raman characterization was done using a confocal Raman microscope (Senterra II, Bruker). Flakes were optically identified and Raman spectra at several locations within the flake measured (for each flake we acquired between 6 and 23 individual spectra, depending on the lateral size of the flake) using a long-working-distance 100× objective (Olympus, LMPLFLN). Each individual spectrum was taken using 633 nm wavelength, power of 2 mW, 10 s acquisition time, and 10 coacquisitions. The same conditions were used for the bulk characterization. Spectra were individually analyzed using IgorPRO, fitting Gaussian peaks to the Raman modes in the spectral range of interest.

### Thermogravimetric Analysis

Thermogravimetric analyses (TGA) were performed in a Q500 instrument. The general procedure consisted of a fast heating ramp to 100 °C, followed by a 30 min isothermal. Then, a gradual ramp of 10 °C/min to 1000 °C was carried out, and finally, the system was allowed to cool to 50 °C, with a last isothermal of 5 min. The data sampling interval was 0.50 s/pt. The experiments were performed in air.

### Electron Microscopy Measurements

Morphological characterization of MoS_2_ was preliminarily performed using a T20-FEI Tecnai thermoionic transmission electron microscope (TEM) and an Inspect F-50 Scanning Electron Microscope, (SEM FEI, Holland), with the specimen previously coated with a carbon layer. Aberration corrected scanning transmission electron microscopy (Cs-corrected HAADF-STEM) images were acquired using a high angle annular dark field detector in a FEI XFEG TITAN electron microscope operated at 300 kV equipped with a CETCOR Cs-probe corrector from CEOS.

### AFM Analysis

Seven different areas of the sample (separated by tens to hundreds of micrometers) were analyzed by AFM (Ntegra Prima, NT-MDT), in semicontact (dynamic) mode using scanning by sample configuration in ambient conditions and a rectangular silicon cantilever (NSG01, NT-MDT, tip radius 10 nm, nominal spring constant 5.1 N/m, resonance frequency around 150 kHz). In each area, we took images of several flakes which were individually analyzed in size and height (a total of 27 flakes were localized and studied). For each flake, we extracted profiles of all different steps visible in the images using Gwyddion, and measured the step height by adjusting edge height functions.

### XPS Analysis

XPS analysis was performed with an AXIS Supra (Kratos Tech.). The spectra were excited by a monochromatic AlK source (1486.6 eV) run at 15 kV and 15 mA. The photoelectron takeoff angle (angle of the surface with the direction in which the photoelectrons are analyzed) was 90°. For the individual peak regions, a pass energy of 20 eV was used. Adventitious carbon was set as reference at 284.8 eV. Peaks were analyzed with CasaXPS software.

### Electron Transport Measurements

The current–voltage characteristics are measured in a Lakeshore electrical probe-station equipped with a Keithley 2450 digital source-meter unit and a Tenma 72–270 Programmable DC Power Supply (60 V, 3 A).

## Supporting Information Available

Screening of exfoliation conditions (dispersion solvent, prewetting solvent, microwave power, microwave irradiation time, MoS_2_–NMP ratio, and % yield); UV–vis analyses, optical and electronic microscopy analyses; thermogravimetric analysis of bulk and exfoliated MoS_2_, X-ray photoelectron spectroscopy analysis, electron transport measurements (PDF)
