# Supplementary material for: Microwave-Driven Exfoliation of Bulk 2H-MoS2 after Acetonitrile Prewetting Produces Large-Area Ultrathin Flakes with Exceptionally High Yield
Source: ACS Nano. 2023 Mar 14;17(6):5984–93. doi: 10.1021/acsnano.3c00280 (PMC10062031; doi:10.1021/acsnano.3c00280)
Supplement: Supplementary file 1 — nn3c00280_si_001.pdf [file nn3c00280_si_001.pdf]

Supporting information

Microwave-driven exfoliation of bulk 2H-MoS_2 after acetonitrile pre-wetting produces large-area ultrathin flakes with exceptionally high yield

Ramiro Quirós-Ovies[1,2], María Laborda[1], Natalia Martín Sabanés[2], Lucía Martín-Pérez[2], Sara Moreno-Da Silva[2], Enrique Burzurí[2,3], Victor Sebastian[1,4,5,6*], Emilio M. Pérez[2*], Jesús Santamaría[1,4,5]

[1]Instituto de Nanociencia y Materiales de Aragón (INMA), CSIC-Universidad de Zaragoza, Zaragoza 50009, Spain.

[2]IMDEA Nanociencia C/Faraday 9 Ciudad Universitaria de Cantoblanco, 28049 Madrid, Spain.

[3] Departamento de Física de la Materia Condensada and Condensed Matter Physics Center (IFIMAC), Universidad Autónoma de Madrid, 28049 Madrid, Spain

[4]Department of Chemical and Environmental Engineering Universidad de Zaragoza Campus Rio Ebro, 50018 Zaragoza, Spain.

[5]Networking Research Center on Bioengineering, Biomaterials and Nanomedicine (CIBER-BBN), 28029 Madrid, Spain.

[6] Laboratorio de Microscopías Avanzadas, Universidad de Zaragoza, 50018 Zaragoza, Spain

Summary

Screening of exfoliation conditions p2

UV-VIS analyses p6

Optical and electronic microscopy analyses p7

Thermogravimetrical analysis p11

X-Ray Photoelectron Spectroscopy analysis p12

Electron transport measurements p13

References p15

Screening of exfoliation conditions

Different infiltration and dispersion solvents, microwave irradiation parameters (power and irradiation time) and concentrations (from 0.2 mg/mL) were studied (tables S1 and S2). NMP was chosen as a starting point for dispersing the material, due to the well-known capacity of this solvent to stabilize the layers of TMDCs, and in fact it was finally the dispersion solvent chosen.

Different pre-wetting solvents were then tested, finding out that ACN exhibited the best behaviour in terms of being able to infiltrate the space between the MoS_2 layers and produce a sudden evaporation, exfoliating the material. Power of microwave radiation and time of exposure were changed but the best conditions were found with the highest intensity available (300 W) that lead to the fastest heating and best yields. A short time (90'') under this power was enough to exfoliate the material down to few-layered MoS_2.

Table S1. Yields of MW-exfoliated samples of MoS_2, employing NMP as the main solvent and changing the pre-wetting, power-time heating in the microwave, MoS_2-NMP ratio and % yield. In all cases, % yield was calculated after 2000 rpm - 30' centrifugation and filtering supernatants through PTFE, 0.2 µm membranes.

MAIN SOLVENT	Pre-wetting solvent	Power - Time	Ratio (mg/mL)	Yield (%)
NMP	-	300 W - 90 s	1	8
NMP	ACN	300 W - 90 s	1	37
NMP	Acetone	300 W - 90 s	1	22
NMP	Tetrahydrofuran	300 W - 90 s	1	31
NMP	Cyclohexane	300 W - 90 s	1	13
NMP	Toluene	300 W - 90 s	1	13
NMP	*i*-PrOH	300 W - 90 s	1	26
NMP	ACN	300 W - 90 s	10	2
NMP	ACN	300 W - 90 s	6	3
NMP	ACN	300 W - 90 s	3.3	5
NMP	ACN	300 W - 90 s	1.7	18
NMP	ACN	300 W - 90 s	0.2	47
NMP	ACN	15 W - 20'	1	3
NMP	ACN	30 W - 10'	1	2
NMP	ACN	50 W - 5'	1	16
NMP	ACN	75 W - 3'	1	15
NMP	ACN	100 W - 2'	1	21
NMP	ACN	200 W - 90'	1	28

Other dispersing solvents were also tested, keeping ACN as the pre-wetter. Power-time conditions were changed due to the lower boiling point of these other solvents, compared to NMP. Acetone was the only option found to be also suitable for MoS2 exfoliation, and mass-volume ratios were evaluated in this case. UV-Vis spectra of some samples are shown below (Fig. SI 1). Nevertheless, NMP-ACN is still the best combination found for the MW-assisted exfoliation of the material. Acetone-ACN exfoliated samples produced very thin layers (< 4 nm) but with smaller lateral size that the NMP-ACN method, as measured by AFM (Fig SI 2).

Table S2. Yields obtained for MW-exfoliated samples of MoS_2, employing different solvents, power-time heating in the microwave, and MoS_2-Solvent ratio and % yield. In all cases, yield was calculated after 2000 rpm -30' centrifugation and filtering supernatants through PTFE, 0.2 µm membranes.

MAIN SOLVENT	Pre-wetting solvent	Power - Time	Ratio (mg/mL)	Yield (%)
ACN	-	75 W - 30 S	1	7
ACN	ACN	75 W - 30 s	1	8
ACN	ACN	50 W - 60 s	1	6
Acetone	-	50 W - 30 s	1	5
Acetone	ACN	50 W - 30 s	1	16
Acetone	ACN	30 W - 60 s	1	10
Acetone	ACN	50 W - 30 s	10	3
Acetone	ACN	50 W - 30 s	6	4
Acetone	ACN	50 W - 30 s	3.3	4
Acetone	ACN	50 W - 30 s	3	10
Acetone	ACN	50 W - 30 s	2	10
Acetone	ACN	50 W - 30 s	1.7	19
Acetone	ACN	50 W - 30 s	0.6	24
Acetone	ACN	50 W - 30 s	0.2	31
CHCl3	-	50 W - 30 s	1	3
CHCl3	ACN	50 W - 30 s	1	8
CHCl3	ACN	30 W - 60 s	1	5
i-PrOH	-	75 W - 30 s	1	1
i-PrOH	ACN	75 W - 30 s	1	5
i-PrOH	ACN	50 W - 60 s	1	2
THF	-	50 W - 30 s	1	0
THF	ACN	50 W - 30 s	1	0
THF	ACN	30 W - 60 s	1	0
Cyclohexane	-	75 W - 30 s	1	0
Cyclohexane	ACN	75 W - 30 s	1	0
Cyclohexane	ACN	50 W - 60 s	1	0

Several combinations of acetonitrile as pre-wetting solvent with different microwave-absorbing solvents were tested. At the beginning, ACN was also exchanged by acetone, THF, $CHCl_3$, toluene, cyclohexane and isopropyl alcohol, to prove their capability to introduce between the MoS_2 layers. In all cases, yields obtained were worse than in the analogous ACN reaction and, furthermore, the quality of the material was considerably inferior: thicker flakes and/or smaller layers (Fig. SI 11).

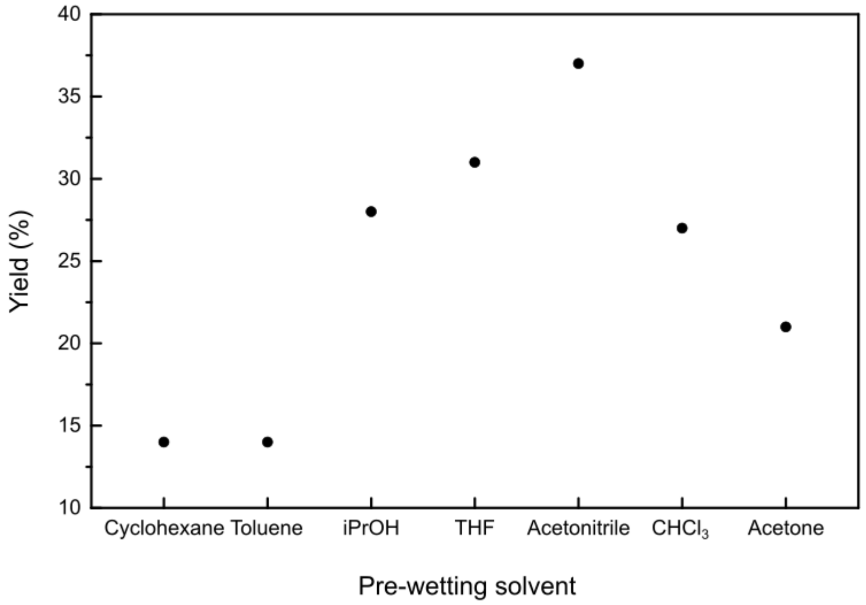

Fig SI 1. Yield obtained for MW-exfoliated samples of MoS_2, employing NMP as the main solvent and seven different options as pre-wetting agents.

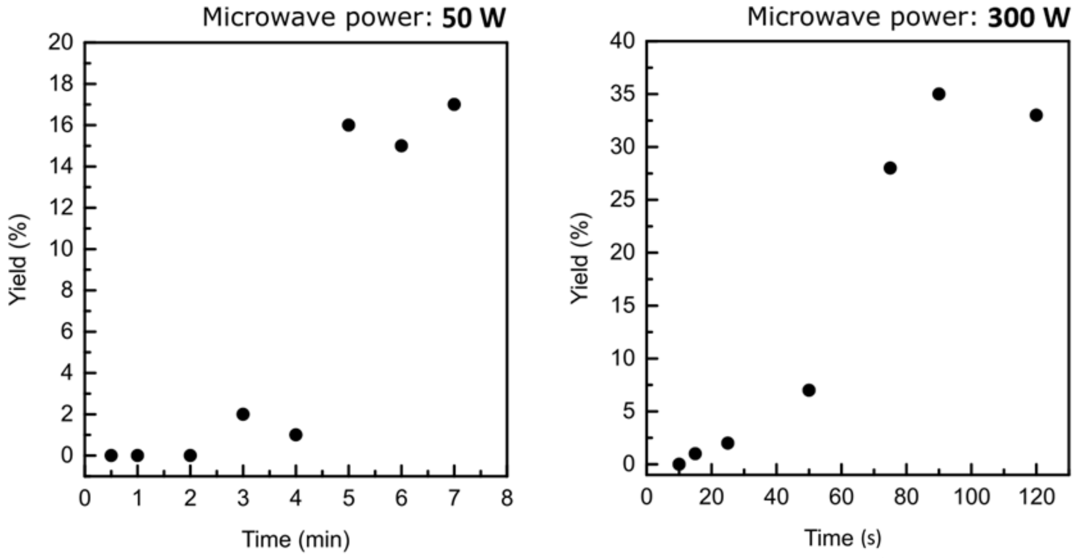

Fig SI 2. Exfoliation *yield of NMP-ACN exfoliated samples vs heating time at two different microwave heating power: 50 W and 300 W.*

UV-VIS analyses

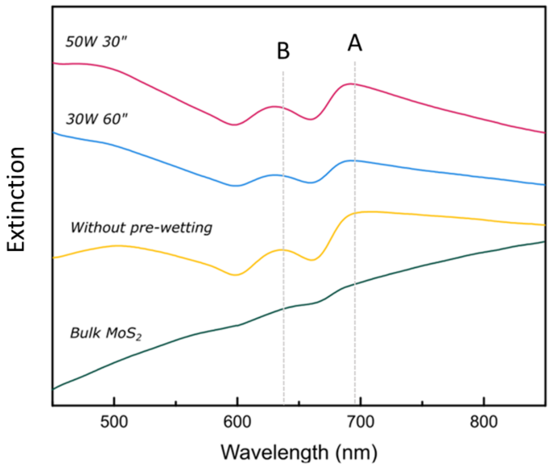

Fig SI 3. UV-Vis spectra of Acetone-ACN exfoliated samples, compared to bulk MoS_2 and dispersed in NMP in all cases. Exfoliated materials were centrifuged at 2000 rpm – 30' and 1 mg/mL was the MoS_2-NMP ratio employed in the four examples.

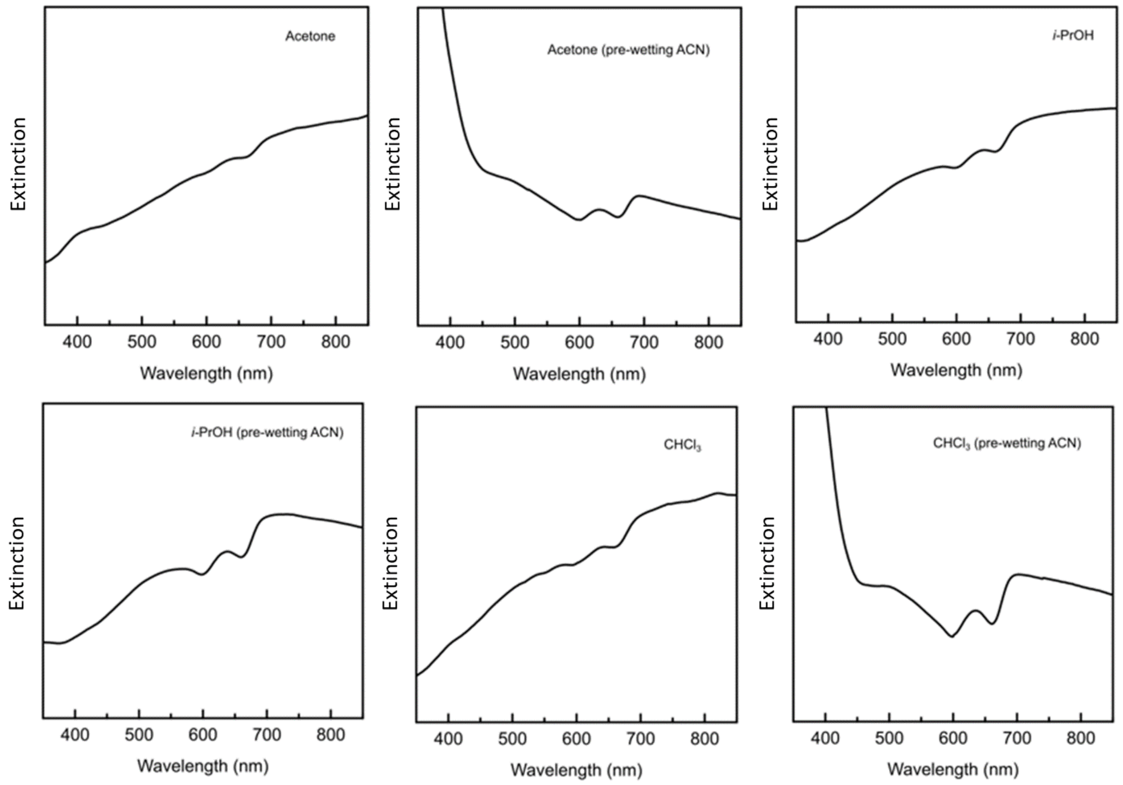

Fig SI 4. UV-Vis spectra of MoS_2 after MW-heating and 2000 rpm – 30' centrifugation, with some of the solvents tested, with and without pre-wetting.

Optical and electronic microscopy analyses

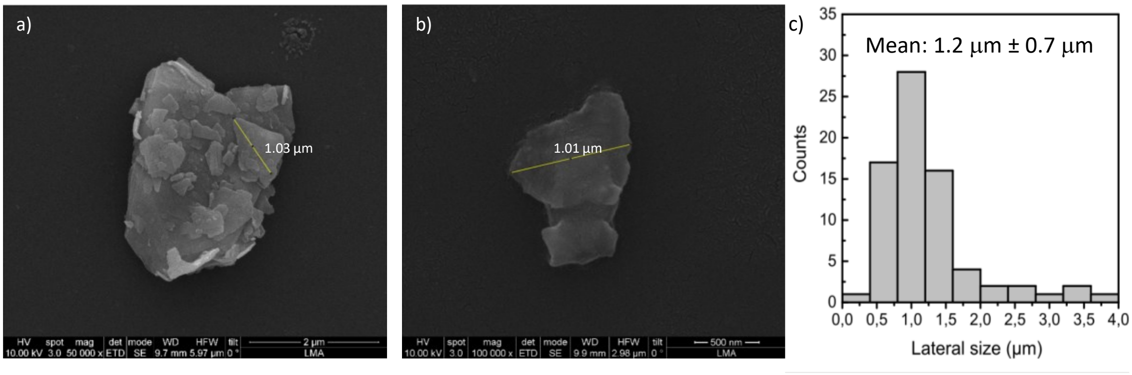

Fig SI 5-SEM Images of a) bulk MoS_2 ,b) MW-exfoliated MoS_2 and c) Lateral size histogram (N = 74)

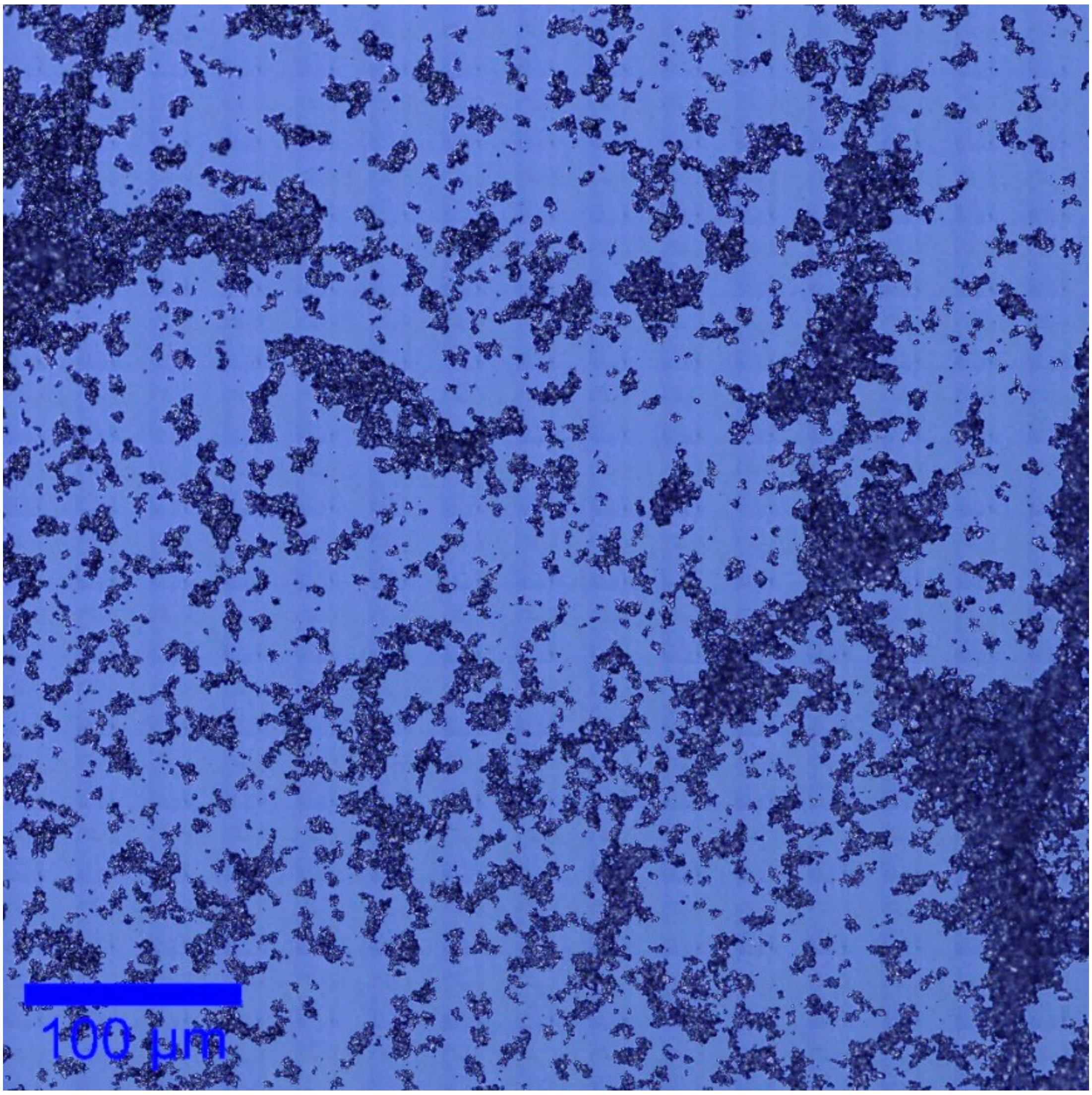

Fig SI 6. Optical image of bulk MoS_2, constructed by adding hundreds of images of the whole sample. A thick material (obscure under visible light) is constantly seen. Sample was prepared by drop-casting the ACN-dispersed material onto a SiO_2 substrate and allowed to dry under air.

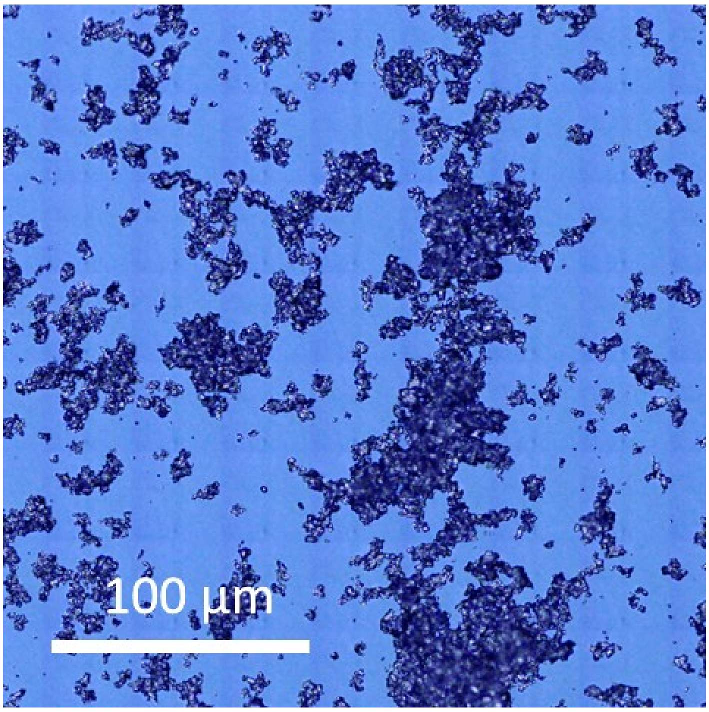

Fig SI 7. Optical image of bulk MoS_2, amplified.

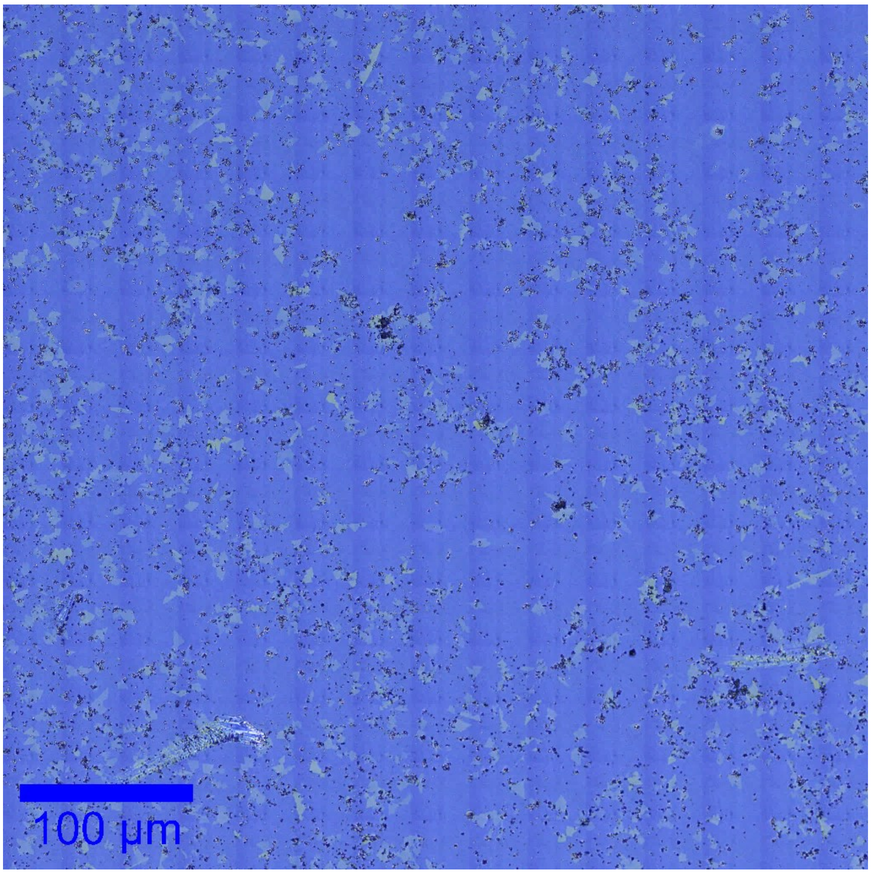

Fig SI 8. Optical image of exfoliated MoS_2 (NMP-ACN, 300W 90'', 2000 rpm 30'), constructed by adding hundreds of images of the whole sample. Extremely thin (almost transparent in some cases) and laterally big layers are observed all over the sample. Sample was prepared by drop-casting the ACN-dispersed material onto a SiO_2 substrate and allowed to dry under air.

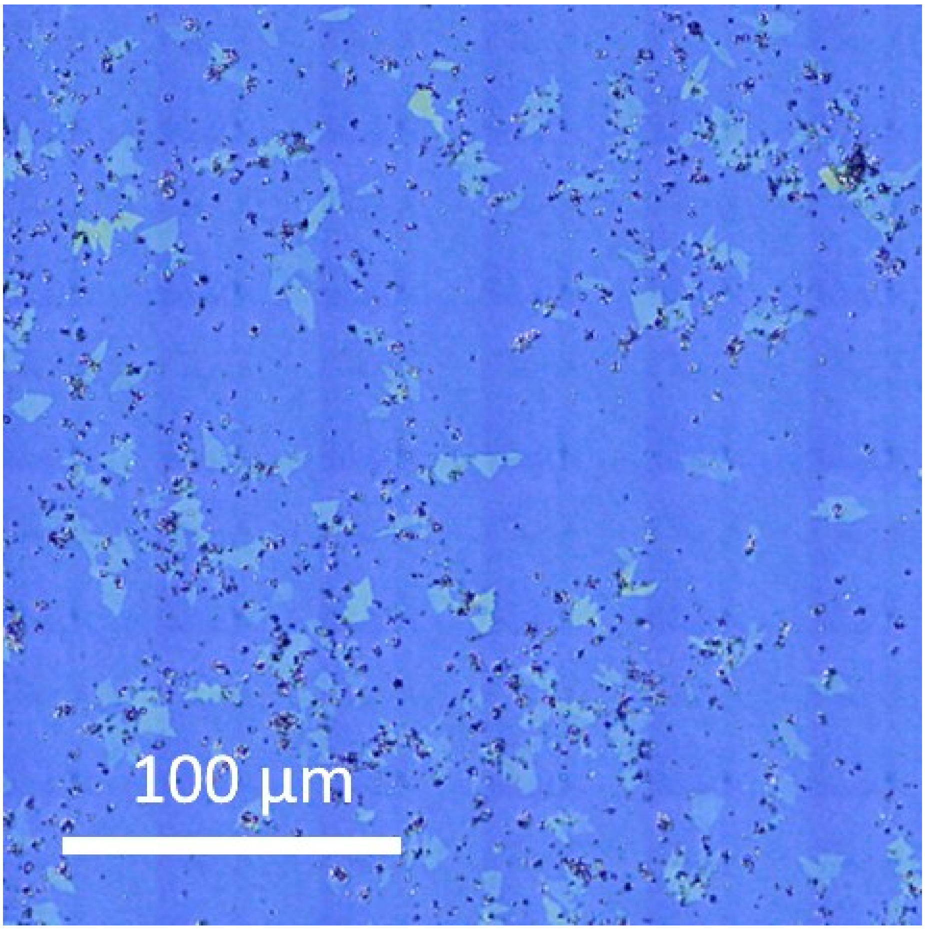

Fig SI 9. Optical image of exfoliated MoS_2, amplified.

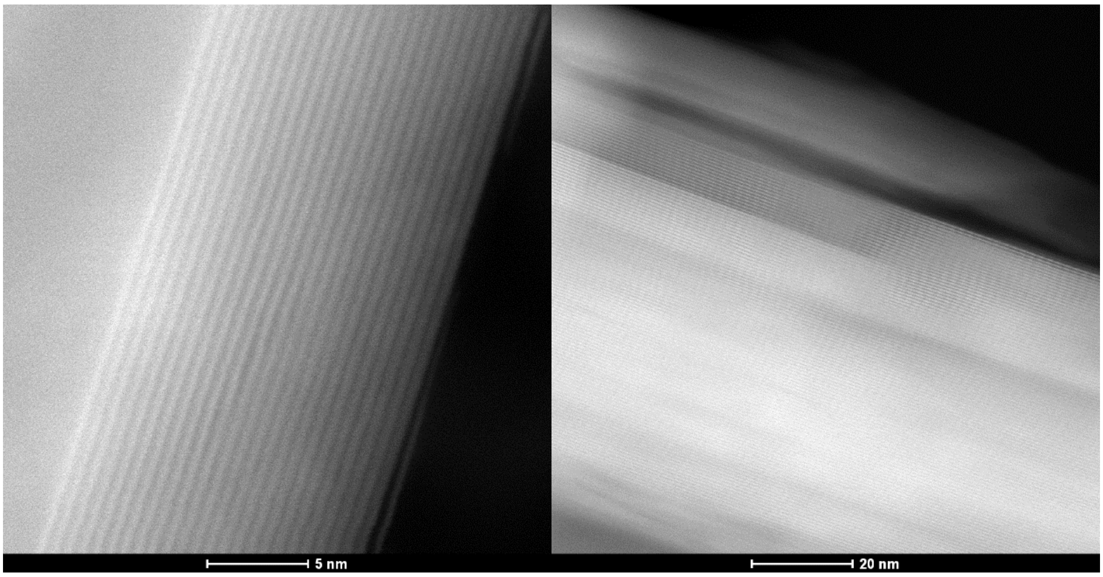

Fig SI 10. HAADF-STEM images of bulk MoS_2 dispersed in ACN and then drop-casted onto holey carbon TEM grid. Thick layers are generally observed all over the sample.

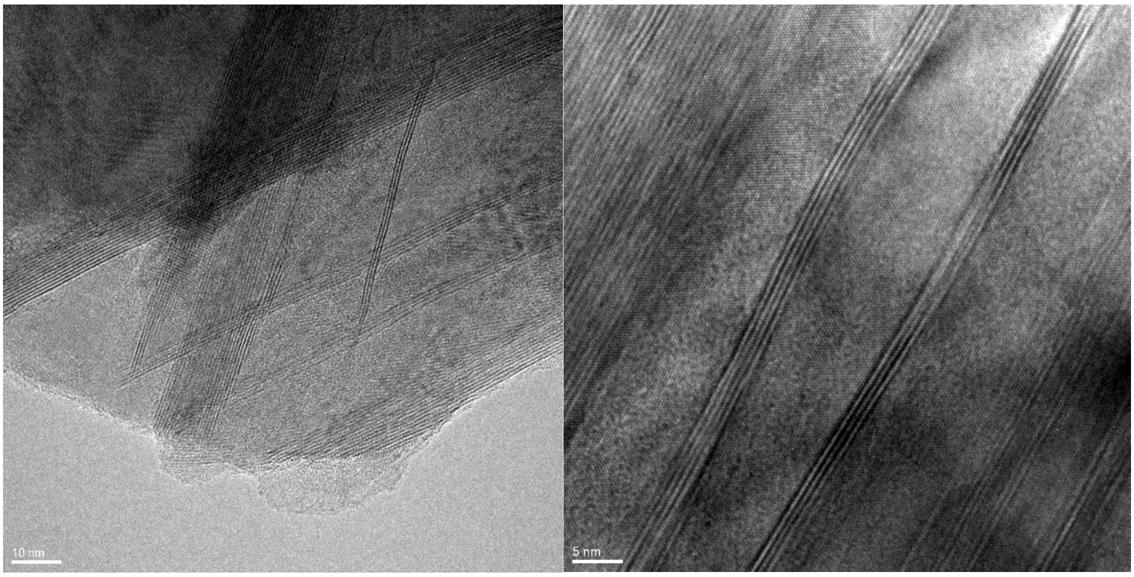

Fig SI 11.HR-TEM images of exfoliated MoS_2 with NMP (ACN pre-wetting). Despite the re-stacking during drop-casting for sample preparation, thin flakes of the material (<5 layers) are repeatedly seen.

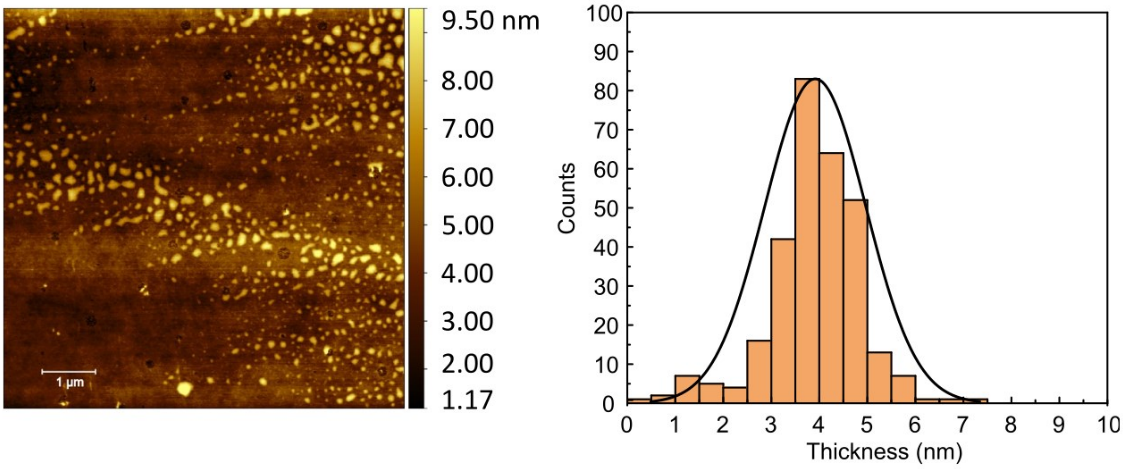

Fig SI 12. AFM image and statistical analysis of the height of the flakes obtained with Acetone-ACN exfoliation, 50W-30" MW-heating, 1 mg/mL ratio and 2000 rpm 30' centrifugation. The sample was prepared drop-casting the supernatant after centrifugation onto a SiO_2 substrate.

Table SI 3. Height, number of layers and lateral size statistics of the MW-exfoliated MoS_2 flakes obtained after AFM (thickness) and SEM (size) analysis.

Parameter	N total	Mean	Standard Deviation	Minimum	Median	Maximum
Thickness, (nm) -AFM	498	4.3	2.5	0.4	4.0	26.0
Number of layers-AFM	498	4.0	2.3	0.4	3.6	23.6
Lateral Size (μm)-SEM	74	1.2	0.7	0.3	1.0	3.9

Thermogravimetrical analysis

Thermogravimetrical analysis of bulk and exfoliated MoS_2 was performed to evaluate the thermal stability and facility to be oxydized. No significant differences were found, but an apparent increased stability in the exfoliated sample (the main losses appear at higher temperatures) and, above all, a notable increased homegeneity is exhibited by the MW-exfoliated MoS_2 (only one obvious loss is observed at 850ºC)

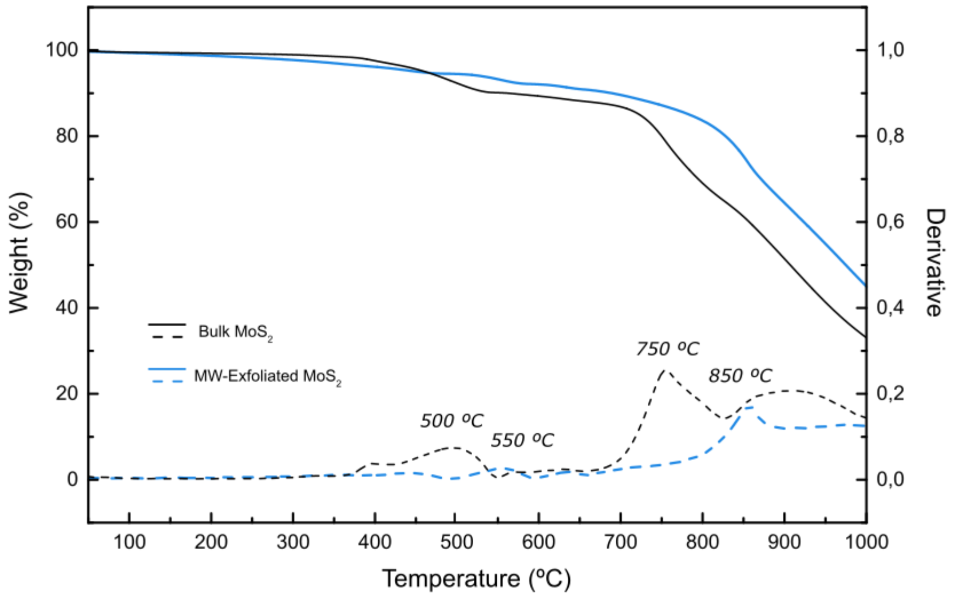

Fig SI 13. TGA analysis of bulk (black) and MW-exfoliated (blue) MoS_2. The exfoliated sample corresponds to NMP-ACN, 300W 90" and 1 mg/mL exfoliation, after 2000 rpm 30' centrifugation.

X-Ray Photoelectron Spectroscopy analysis

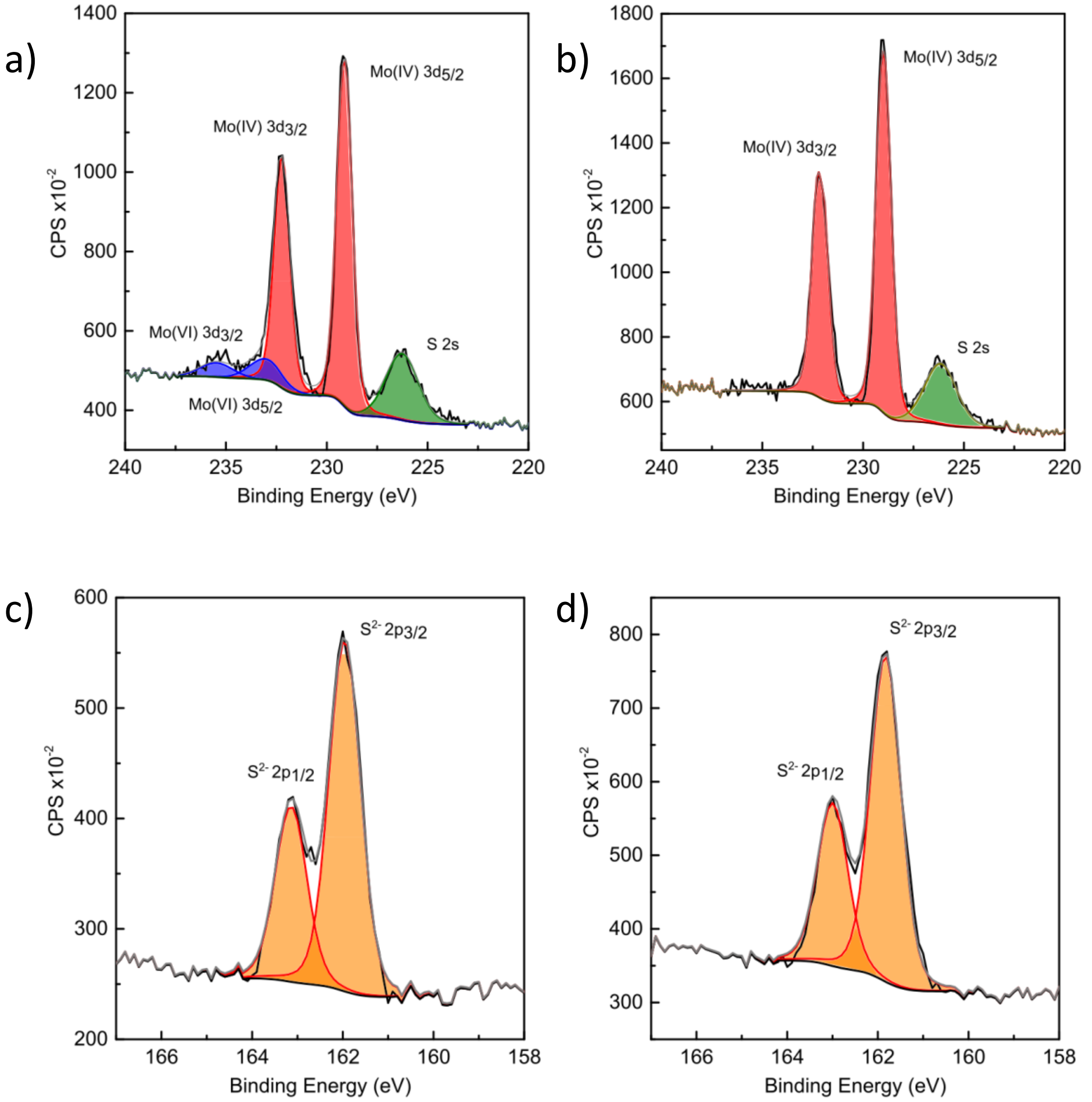

Fig SI 14. X-Ray Photoelectron Spectroscopy (XPS) analysis of a) bulk MoS_2 Mo 3d, b) MW-exfoliated MoS_2 Mo 3d, c) bulk MoS_2 S 2p and d) MW- exfoliated MoS_2 S 2p.

Electron transport measurements

The electrical properties of exfoliated MoS_2 flakes are explored in solid-state electronic devices. MoS_2 flakes are placed into metallic electrodes directly from solution by dielectrophoresis. In short, a NMP micro-droplet containing the dispersed MoS_2 flakes is deposited on top of two Au electrodes (source-drain) on a Si/SiO_2 substrate. See Methods for nanofabrication details. Simultaneously, an AC voltage is applied between the electrodes (f = 1 MHz, V = 10 V, t = 5 min) that generates an AC electrical field. The suspended nanoflakes are polarized by the electric field, attracted and aligned towards the area with a larger electrical gradient; *i.e.* the gap between the electrodes. NMP is thereafter blown with nitrogen and the device kept at 10^{-6} mbar to remove NMP residues. See Ref.[1,4] for a detailed explanation of the dielectrophoresis-based positioning of nano-objects. Figures SI 15 a,b show the Scanning Electron Microscopy (SEM) images of two representative MoS_2-FETs fabricated by dielectrophoresis. A single MoS_2 flake bridges the gap between the Au source and drain electrodes.

Figure SI 15 c,d shows the current I – Voltage V_{sd} characteristics measured on two representative devices. The first sign of electrical contact between flake and electrodes is the sharp increment of the conductance (red curve) when compared with the empty device before deposition of the flake (black curve). Current values increase to the nanoampere range, corresponding to GΩ-range resistance, that is of the order of those reported in the literature for thin flakes.[2,3] These values may largely vary depending on the dimensions of the device, the thickness of the flake, strain and the contact with the electrodes.

The shape of the I-V_{sd} characteristics slightly deviates from the ohmic behavior signaling the presence of Schottky barriers at the interface between MoS_2 and Au electrodes, that may lead to rectifying and diode-like behavior, as reported before.[2] Figure SI 15 e shows an example of this rectifying behavior.

Finally, the Si substrate has been used as back-gate electrode to apply a gate voltage V_{bg} to the MoS_2 flakes (Figures SI 15 e,f). A certain gate dependence is observed in line with previous reports for MoS_2,[5] which could be also associated with tuning of the Schottky barriers.

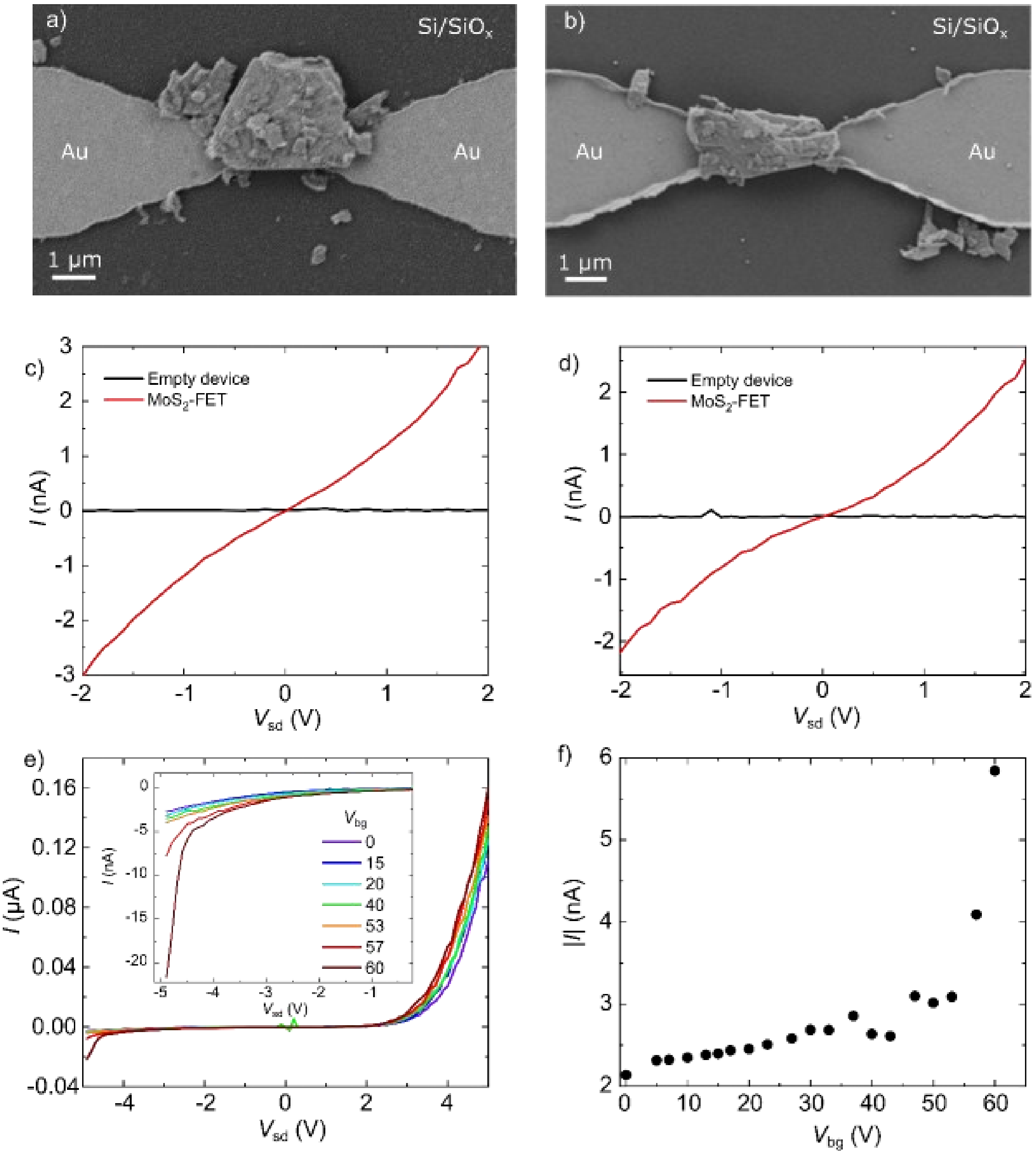

Figure SI 15. (a,b) Scanning Electron Microscopy (SEM) image of two representative MoS_2-FET devices. Individual MoS_2 flakes bridge the gap between gold source and drain electrodes. (c,d) Current I – Voltage V_{sd} characteristics measured in two MoS_2-FET devices. The IV_{sd} characteristics show an almost ohmic behavior with a slight deviation as result of the formation of Schottky barriers between MoS_2 and electrodes. (e) IV_{sd} characteristics of a MoS_2-FET device showing rectifying behavior. The different colors correspond to different back-gate voltage V_{bg} applied. The inset shows a zoom on the negative bias region. (f) Absolute value of the current as a function of the back-gate voltage measured at V_{sd} = -4.5 V. A certain tuning of MoS_2 and the Schottky barriers can be observed.

References-

(1) Burzurí, E.; Vera-Hidalgo, M.; Giovanelli, E.; Villalva, J.; Castellanos-Gomez, A.; Pérez, E. M. Simultaneous Assembly of van Der Waals Heterostructures into Multiple Nanodevices. *Nanoscale* **2018**, *10* (17), 7966–7970. https://doi.org/10.1039/C8NR01045E.

(2) Quereda, J.; Palacios, J. J.; Agräit, N.; Castellanos-Gomez, A.; Rubio-Bollinger, G. Strain Engineering of Schottky Barriers in Single- and Few-Layer MoS2 Vertical Devices. *2D Mater.* **2017**, *4* (2), 021006. https://doi.org/10.1088/2053-1583/AA5920.

(3) Çakıroğlu, O.; Island, J. O.; Xie, Y.; Frisenda, R.; Castellanos-Gomez, A. An Automated System for Strain Engineering and Straintronics of 2D Materials. *Adv. Mater. Technol.* **2022**, 2201091. https://doi.org/10.1002/ADMT.202201091.

(4) Martín-Pérez, L.; Burzurí, E. Optimized Liquid-Phase Exfoliation of Magnetic van Der Waals Heterostructures: Towards the Single Layer and Deterministic Fabrication of Devices. *Molecules* **2021**, *26* (23), 7371. https://doi.org/10.3390/MOLECULES26237371.

(5) Baugher, B. W. H.; Churchill, H. O. H.; Yang, Y.; Jarillo-Herrero, P. Intrinsic Electronic Transport Properties of High-Quality Monolayer and Bilayer MoS2. *Nano Lett.* **2013**, *13* (9), 4212–4216. https://doi.org/10.1021/NL401916S/SUPPL_FILE/NL401916S_SI_001.PDF.
